# RETRACTION: Comparison Efficacy and Safety of Gemcitabine plus Cisplatin and 5‐Fluorouracil plus Cisplatin for Metastatic Nasopharyngeal Carcinoma: A Meta‐Analysis and Systematic Review

**DOI:** 10.1155/jo/9832316

**Published:** 2026-08-07

**Authors:** Journal of Oncology

RETRACTION: L. Yan, H. Zheng, B. Ren, H. Zhang, H. Gou, L. Dai, “Comparison Efficacy and Safety of Gemcitabine plus Cisplatin and 5‐Fluorouracil plus Cisplatin for Metastatic Nasopharyngeal Carcinoma: A Meta‐Analysis and Systematic Review,” *Journal of Oncology* (2022): 1‐10, https://doi.org/10.1155/2022/7233559.

The above article, published online on 16th July 2022 in Wiley Online Library (https://wileyonlinelibrary.com), has been retracted by agreement between the Journal Editor of “Cancer,” Suresh S. Ramalingam, and John Wiley & Sons Ltd.

The retraction has been agreed following the investigation of concerns initially raised by a reader on PubPeer [1]. The authors included non‐Randomised Control Trials in Section 3.2, despite declaring the cited studies as being Randomised Control Trials. The systematic review also incorrectly uses the RoB2 instrument for RCTs for non‐RCTs. The resulting graphic makes it appear as if all but one of the studies are randomized.

The results are therefore considered unreliable, and the article is retracted.

## References

[1] Matthias Perleth , Comparison Efficacy and Safety of Gemcitabine plus Cisplatin and 5-Fluorouracil plus Cisplatin for Metastatic Nasopharyngeal Carcinoma: A Meta-Analysis and Systematic Review, PubPeer. (September 2025) https://pubpeer.com/publications/3879864583E7B9ACB8F52BBB39CB4A. 10.1155/2022/7233559 PMC9308559 35880116

